# Solid Lipid Nanoparticles for Drug Delivery: Pharmacological and Biopharmaceutical Aspects

**DOI:** 10.3389/fmolb.2020.587997

**Published:** 2020-10-30

**Authors:** Sebastián Scioli Montoto, Giuliana Muraca, María Esperanza Ruiz

**Affiliations:** ^1^Laboratorio de Investigación y Desarrollo de Bioactivos, Departamento de Ciencias Biológicas, Facultad de Ciencias Exactas, Universidad Nacional de La Plata, La Plata, Argentina; ^2^Consejo Nacional de Investigaciones Científicas y Técnicas, Buenos Aires, Argentina; ^3^Instituto Nacional de Medicamentos (INAME, ANMAT), Buenos Aires, Argentina

**Keywords:** clinical trials, drug delivery, nanostructured lipid carriers, nanotoxicity, pharmacokinetics, pharmacodynamics, routes of administration, solid lipid nanoparticles

## Abstract

In the golden age of pharmaceutical nanocarriers, we are witnessing a maturation stage of the original concepts and ideas. There is no doubt that nanoformulations are extremely valuable tools for drug delivery applications; the current challenge is how to optimize them to ensure that they are safe, effective and scalable, so that they can be manufactured at an industrial level and advance to clinical use. In this context, lipid nanoparticles have gained ground, since they are generally regarded as non-toxic, biocompatible and easy-to-produce formulations. Pharmaceutical applications of lipid nanocarriers are a burgeoning field for the transport and delivery of a diversity of therapeutic agents, from biotechnological products to small drug molecules. This review starts with a brief overview of the characteristics of solid lipid nanoparticles and discusses the relevancy of performing systematic preformulation studies. The main applications, as well as the advantages that this type of nanovehicles offers in certain therapeutic scenarios are discussed. Next, pharmacokinetic aspects are described, such as routes of administration, absorption after oral administration, distribution in the organism (including brain penetration) and elimination processes. Safety and toxicity issues are also addressed. Our work presents an original point of view, addressing the biopharmaceutical aspects of these nanovehicles by means of descriptive statistics of the state-of-the-art of solid lipid nanoparticles research. All the presented results, trends, graphs and discussions are based in a systematic (and reproducible) bibliographic search that considered only original papers in the subject, covering a 7 years range (2013-today), a period that accounts for more than 60% of the total number of publications in the topic in the main bibliographic databases and search engines. Focus was placed on the therapeutic fields of application, absorption and distribution processes and current efforts for the translation into the clinical practice of lipid-based nanoparticles. For this, the currently active clinical trials on lipid nanoparticles were reviewed, with a brief discussion on what achievements or milestones are still to be reached, as a way of understanding the reasons for the scarce number of solid lipid nanoparticles undergoing clinical trials.

## Introduction

For many years, lipid materials that are solid at room temperature have been used in the pharmaceutical industry for the preparation of different types of formulations such as emulsions, lotions, ointments and suppositories, among others (de Blaey and Polderman, 1980). Due to the high affinity of the lipid-rich intercellular space of the stratum corneum for this kind of materials, they have been most commonly used as inert ingredients in topical medications, but lipids (both solid or liquid at room temperature) are also regular constituents of other enteral and parenteral formulations, like soft/hard capsules or parenteral emulsions (Feeney et al., 2016).

Nanoscience, on the other hand, arose initially from the field of physics and electronic engineering, to rapidly impact other scientific areas, such as biology, biochemistry, and medicine, where the size range of nanoparticles (NPs) has historically been associated with the so-called *colloids* (Hauser, 1955). Colloidal systems are dispersion of particles (very large molecules or molecule aggregates) of intermediate size between molecules in solution and particles in coarse suspension, and it has been almost 100 years since a *colloidal* size range of 1–1000 nm was proposed (le Chatelier, 1919), which is still accepted today (McNaught and Wilkinson, 1997).

Hence, the novelty that NPs brought to the biomedical and therapeutic fields was not their size, but a radical change in the prevailing therapeutic paradigm: a designed, tailored, functional or at least protective system, usually carrying a drug, that could reach the systemic circulation of the patient along with the drug. In other words, due to their size, nanovehicles brought down the classical concept that only drugs dissolved in biological fluids can be absorbed and/or distributed through the body.

When back in the 90s Müller et al. (2002) proposed the term *solid lipid nanoparticles* (SLN ®), as well as *nanostructured lipid carriers* (NLC®), it seemed like a natural idea: to combine the advantageous characteristics of NPs (mostly metallic and polymeric at that time) with those of lipid-based parenteral emulsions, based on non-toxic and biodegradable lipid components (Schwarz et al., 1994). These lipid NPs were promoted as a safer option compared to other nanosystems; they are constituted of a solid matrix that would allow the controlled release of the drug, but being more stable (and certainly cheaper) than phospholipid-based liposomes developed so far (Martins et al., 2007).

If lipid NPs were up to the expectations, is what remains to be determined. With that in mind, this review presents an overview of the investigations regarding SLN and NLC for drug delivery applications, and a descriptive statistical analysis of the field from 2013 until today. No size restrictions have been imposed on the systems considered, and the nano-classification proposed by the authors is maintained. Consequently, despite that all the reviewed nanovehicles belong to the colloidal size range mentioned before, those closer to the upper limit could be better addressed as microparticles, which have been in the pharmaceutical market for several years now (Siepmann and Siepmann, 2006).

Our work presents an original point of view, by addressing the biopharmaceutical aspects of these nanovehicles by means of trends and descriptive statistics covering the last 7 years of research in the field. In gathering and presenting the information, focus was placed on the therapeutic fields of application, pharmacokinetic aspects, safety issues, toxicological concerns and current efforts for the translation into the clinical practice of lipid-based NPs. We believe it will be a valuable read for all those researchers interested in knowing what therapeutic challenges are being addressed through the use of SLN, and what remains to be done.

### Our Bibliographic Search

At present, we are witnessing a huge expansion of scientific knowledge, with countless work groups researching common themes, collaboratively or individually, throughout all countries all over the world. This makes it virtually impossible to review topics in a comprehensive manner, that is, covering everything published to date with respect to a given topic. Limiting the information reviewed is thus imperative, with the inevitable risk of falling into involuntary biases regarding the information sampling.

Therefore, in order to perform a search as objective as possible, the following criteria were set:

• Original publications in English dating from the last 7 years: this meant excluding from the systematic search the review articles and limiting the search to original works published since 2013, inclusive. Although it is true that there is a lot of information prior to that date, the analysis of year-by-year statistics of academic databases and search engines reveals that, of the total number of publications retrieved when searching for the phrase “solid lipid nanoparticles” in the title, more than 60% correspond to the period 2013-2020. In particular, publications from 2013 to date were 1600 out of 2630 in Google Scholar (60.8%), 944 out of 1264 in PubMed (74.7%), and 1300 out of 2111 in Scopus (61.6%), which is remarkable considering that the total SLN/NLC publication period is 25 years [the first references date from Maaßen et al. (1993); Muller et al. (1993)].
• R&D publications focused on the application of lipid nanosystems for the delivery of drugs, excluding merely technological developments without any biological or biorelevant assay. For this, the following keywords were included in the search (with the “OR” connector): drug delivery, in vivo, cell, cells, pharmacokinetic, pharmacokinetics.

Despite Google Scholar retrieved the largest number of publications, Scopus was selected to perform the final search due to its more versatile advanced search interface, and the possibility to download the search results. At the time of the writing of this work, this search yielded 371 scientific articles, which constitute the database on which the descriptive statistics and trends presented in the following sections are based.

### The Tiny Big Universe of Lipid Nanoparticles

When firstly developed, SLN were presented as tiny and spherical particles, made of solid lipids at room temperature, that may be thought as perfect crystal lipid matrices, able to accommodate a drug or other molecules between fatty acid chains (Puri et al., 2009). Nowadays, however, it is known that this is not necessarily true in all cases, since disc-like shape or flat ellipsoidal geometry have also been described (Mazuryk et al., 2016; Shah et al., 2019). Moreover, the loaded drug may be attached mostly to the carrier matrix surface instead of being embedded into the solid core (Pink et al., 2019; Shah et al., 2019).

Almost 10 years after SLN introduction, a second generation of lipid NPs, the nanostructured lipid carriers (NLC) appeared (Muller et al., 2002). Considered as an advanced version of SLN, NLC incorporate into their structure small amounts of liquid lipids at room temperature (oils), to produce structural rearrangements of the matrix. By that time, it was observed that the maturation of the crystalline structure that SLN exhibit along the time often results in the expulsion of the incorporated drug to the surrounding medium (Mehnert and Mäder, 2001). The oils in NLC act by reducing the crystalline degree of the lipid core of SLN, thus avoiding the expulsion of the drug from the matrix and increasing the drug loading capacity and physical and chemical long-term stability (Müller et al., 2002). The highly-ordered crystalline structure of the lipids in a SLN has been recently studied by Pink et al. (2019) whose work provides a detailed description of the internal and external structure of SLN.

A detailed description of the materials and methods used for the synthesis of SLN / NLC is beyond the scope of this review, and can be found elsewhere (see, for example, Geszke-Moritz and Moritz, 2016; Gordillo-Galeano and Mora-Huertas, 2018; Jain and Thareja, 2020). However, it is worth highlighting that, being strongly hydrophobic, lipidic NPs in aqueous environments are very low hydrated or no hydrated at all, and thus they are not able to be spontaneously dissolved or dispersed in water. Therefore, the preparation of these dispersions necessarily implies transferring energy to the system, in order to generate very small particles, with very high specific surface area (Troy, 2000). Regardless the details of each synthesis method, they all share the common feature of an energy-providing step, under the form of ultrasonic waves [probe-type sonication (Haque et al., 2018; Pandya et al., 2018; Scioli Montoto et al., 2018) or ultrasonic bath (Rajpoot and Jain, 2018; Rosière et al., 2018; Chirio et al., 2019), high pressures (de Jesus et al., 2013; Küçüktürkmen and Bozkır, 2018; Wang et al., 2018), high speed homogenization (Nakhlband et al., 2018; Sathya et al., 2018; Youssef et al., 2018), or even microwaves (Shah et al., 2016a)]. The comparative performance of the two most commonly applied methods for lipid NPs preparation, hot homogenization and high pressure homogenization, was evaluated in the first studies within the field (Schwarz et al., 1994).

**FIGURE 1:**
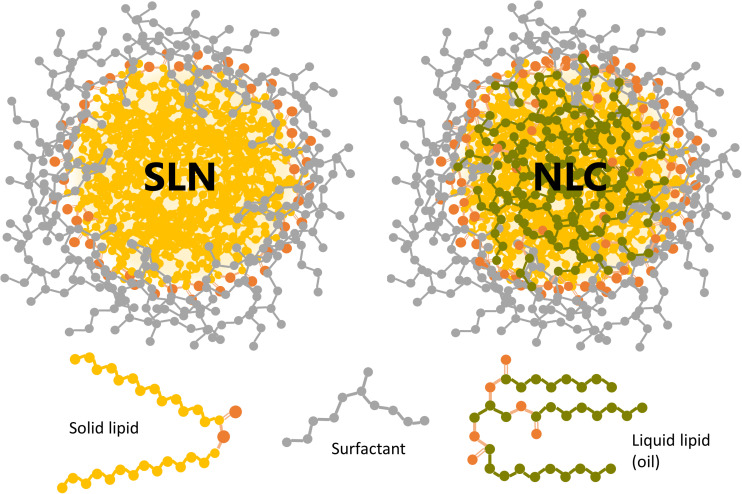
Schematic representation of a SLN and a NLC sterically stabilized with a neutral surfactant (gray). The oxygen atoms in the liquid and solid lipids are shown in orange. Drug molecules are not depicted since they may be located inside the lipid core and/or attached to the outer shell.

On the other hand, besides the energy that must be transferred to the system to *create* the particles, it is also necessary to implement other technological strategies in order to *maintain* the large surface area exposed by the dispersed NPs. Suspended in aqueous media, lipid NPs constitute a lyophobic dispersion (i.e., NPs have no affinity for the dispersing medium), and thus intrinsically unstable. The most stable state of the lyophobic colloids contains the dispersed phase aggregated in large crystals or droplets, to minimize the specific surface area and, hence, the interfacial free energy (Leite and Ribeiro, 2012). To prevent this aggregation process (*coalescence*), the particles must be electrostatically and/or sterically stabilized (Keck et al., 2014; Kovačević et al., 2014). Figure 1 shows a schematic representation of a SLN and a NLC sterically stabilized with a neutral surfactant.

The superficial charge of lipid NPs is mostly determined by the materials used for their synthesis, and the pH of the surrounding medium. The Z potential (ζ) is the electric potential at the slipping (or shear) plane, i.e., the potential difference between the stationary double layer of fluid that surrounds a colloidal particle in suspension and any point in the surrounding liquid medium. It is a measure of the surface charge of the particle, and thus it is directly related to the charge exhibited by the lipid or surfactant of the nanosystem, at the pH value of the formulation (Cheng and Lee, 2016).

The ζ required to stabilize the lipid NPs dispersion only by electrostatic repulsion is usually accepted to be ±30 mV or higher, to assure enough repulsion of nearby NPs in the suspension (Kovačević et al., 2014). Negative values of ζ are achieved when particles are formulated with negatively charged components at the pH of the formulation, like stearic acid (Liu et al., 2017), sodium taurocholate (Rosière et al., 2018) or 1-Oleoyl-glycero-3-phosphate sodium salt (Abd-Rabou et al., 2018), among others. On the contrary, positively charged starting materials are needed to produce lipid NPs with ζ values greater than zero, such as stearylamine (Costa et al., 2018), quaternary ammonium lipids (Doktorovova et al., 2018; Küçüktürkmen and Bozkır, 2018; Wu et al., 2018) or chitosan coatings (Liu et al., 2017; Vijayakumar et al., 2017). Nevertheless, the majority of lipid components (as well as surfactants) currently used to formulate SLN/NLC are neutral, with the two most common being ester (e.g., glycerides) and ether (e.g., Tween, Poloxamer, Brij) functions. Lipid NPs based on these materials tend to present ζ values slightly or moderately negative (between -30 and -3 mV).

The small absolute values of ζ are not enough to prevent the coalescence of the NPs, which need to be further stabilized by steric repulsion. To do so, hydrophilic polymers and/or surfactants are included in the formulation. These compounds tend to adsorb onto the particles surface and project their polar residues to the surrounding aqueous medium, thus preventing the NPs to get too close so that the attractive forces predominate (Luo et al., 2015).

It is not easy, however, predicting the effect that the NPs composition and preparation method will have on the ζ, particle size (PS) and entrapment efficiency (%EE). Systematic approaches like the quality by design (QbD) concepts, strongly related to the pharmaceutical industry, are very useful to comprehensively study and characterize the design space of the formulation. From the 371 articles reviewed, only 48 (nearly 13%) applied this type of analysis.

In terms of the product, QbD tools arise from the recognition that in order to guarantee the quality, it is not enough (nor economically efficient) to verify it in the finished product but has to be incorporated from its design. In the nanotechnology area, and more precisely, the development and preparation of SLN/NLC, this idea means to replace the old development empirical approach (i.e., in an artisanal way) by a more systematic one, based on the experimental design and the statistical analysis of the results (ICH, 2009).

To do so, it is usually convenient to start with fractional factorial designs, which allow to study multiple variables at the same time with the smallest number of runs: while a full factorial design requires 2^k^ experiments or runs to study the effect of k factor at 2 levels (without replicates), the $\frac{1}{4}$ fraction of this design allows estimating main effects with 2^k–2^ runs. The decrease in the number of runs (i.e., in degrees of freedom), inevitably implies a loss of information, but fractional designs are ideal preliminary designs, to study several factors at a time with focus on their main effects, as generally happens during the design of products and processes (Montgomery, 2017).

Once the more relevant factors are identified, a minor number of them are studied with more details [i.e., more levels, so that the “curvature” in the response function can be addressed) in the optimization stage. For this, response surface methodologies (RSM) are usually employed (although other statistical techniques may apply, see for example (Amasya et al., 2019)]. RSM are generated from designs where factors are studied in more than two level (n levels), such as full factorial (n^k^) designs or more efficient ones like the central composite or Box-Behnken designs. This type of designs allows to find functional relationships among studied responses (quality attributes, such as particle size or ζ) and factors, like the amount of lipid, the synthesis time or temperature, among others.

The above mentioned is particularly relevant in SLN/NLC area, since even with the experience accumulated in these years, very few trends are predictable. Perhaps the only example is the positive relationship between the amount of lipid and the particle size, which is verified in almost all the cases and independently of the drug and preparation method: all the review articles that include the study of particle size as a function of the lipid amount found a direct relationship between them, at least in part of the studied range, if not in all. However, these results must be interpreted carefully since the existence of interactions among factors can cause this relationship to be modified according to the levels of the other factors. It is not unusual that at higher surfactant concentrations, the effect of the amount of lipid over the particle size is minor or null (Cacicedo et al., 2019; Rajpoot and Jain, 2019). The presence of interactions among factors and their magnitude can only be studied by means of designs of several crossed factors, such as those mentioned before, being insufficient the individual or univariate optimization of the responses in function of each process attribute or parameter (Montgomery, 2017).

Systematization of the preformulation stage through the aforementioned statistical tools allow not only to gain insights in a more efficient manner but also to study several response variables at once. As said before, decreasing the amount of lipid incorporated into the formulation frequently helps to reduce particle size, but has a negative effect on the entrapment efficiency (Kurakula et al., 2016; Talluri et al., 2017; Ahmad et al., 2019). Simultaneous optimization of both responses as a function of the formulation components and/or process operational variables allows to find the optimum compromise solution as well as other possible approaches, such as increasing the energy (frequency, speed) during the synthesis to decrease the particle size without sacrificing entrapment efficiency (Nooli et al., 2017; Dara et al., 2019; Khatri et al., 2019; Patel et al., 2019a), or decreasing the surfactant/lipid ratio (Bhalekar et al., 2017; Pandya et al., 2018; Ahmad et al., 2019).

## A Descriptive Analysis of Therapeutic Application Fields of SLN

**FIGURE 2:**
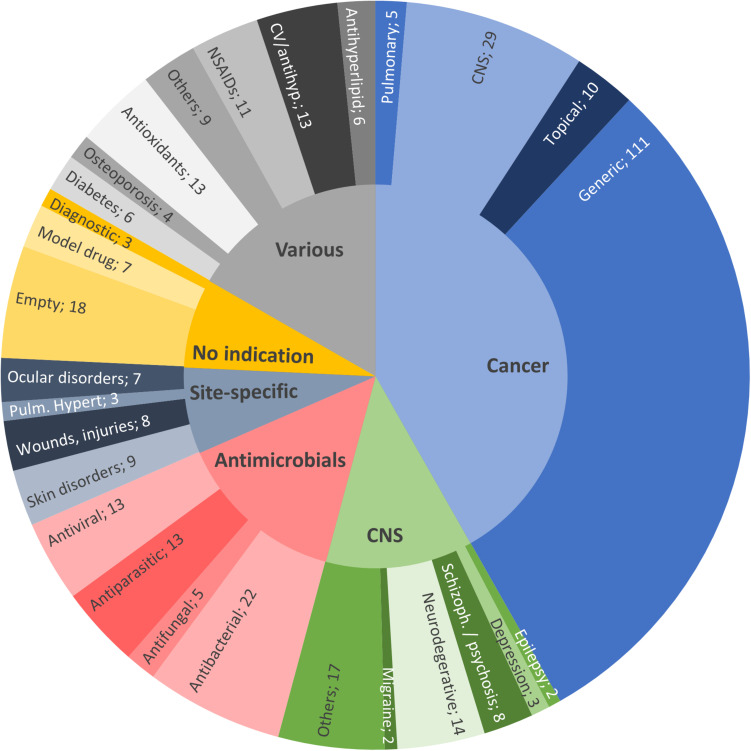
Distribution of the 2013–2020 reviewed publications on SLN/NLC, by therapeutic field: anticancer therapies (41.8%, light blue); antimicrobials (14.3%, pink); CNS diseases, excluding cancer and infection (12.4%, green); site-specific treatments (7.3%, dark blue); various indications (16.7%, gray) and; nanovehicles not intended for any specific therapeutic area (7.5%, yellow).

Figure 2 shows the distribution of publications on SLN/NLC of the last 7 years, grouped by therapeutic fields. It can be seen that, as for other nanosystems, cancer treatment represents the most relevant field of application (Hare et al., 2017). Of the 371 publications surveyed, 41.8% (155) corresponded to anticancer therapies, 14.3% (53) to antimicrobials, 12.4% (46) to the treatment of central nervous system (CNS) diseases and/or disorders (excluding cancer and infection), 7.3% (27) to site-specific treatments, 7.5% (28) to nanovehicles not intended for any specific therapeutic area (i.e., with no indication, including SLN for diagnostic purposes) and the remaining 16.7% (62) comprises drugs for various conditions or diseases (in gray in Figure 2).

In general, the treatment of any disease can be enhanced by the formulation of drugs loaded into lipid-based NPs, mainly due to physicochemical and/or biopharmaceutical aspects, like an improved pharmacokinetic profile, as we will discuss in the next section. However, the distribution displayed in Figure 2 suggests that lipid-based NPs may possess additional advantages in certain specific therapeutic fields.

The large efforts in nanotechnologies focusing on **cancer treatment** is not surprising. Cancer is one of the major public health concerns and is among the leading causes of death worldwide. According to the National Cancer Institute (NCI, NIH), while in 2012 there were 14.1 million new cases (and 8.2 million cancer-related deaths worldwide), it is expected that the number of new cancer cases per year will reach 23.6 million by 2030^1^.

The analysis of the database entries corresponding to SLN/NLC for cancer treatment reveals a great variety of encapsulated drugs, from large lipophilic molecules such as taxanes, to small molecules of higher polarity such as 5-fluorouracil. Even Pt-based chemotherapeutic agents (like the water-soluble drugs cisplatin and oxaliplatin) have been efficiently loaded into SLN, highlighting the versatility of these nanocarriers to encapsulate almost the whole range of chemotherapeutic agents available today.

In addition to the strong reasons for seeking new strategies for cancer therapies, cancerous tissues possess unique characteristics that make the choice of nano-based drug delivery especially interesting. The high rate of tumor growth leads to abnormal angiogenesis, with abundant fenestrations and large gaps between endothelial cells, as well as deficient lymphatic drainage in the area (von Roemeling et al., 2017). Combined, these characteristics lead to the accumulation, only based on the size (i.e., passive targeting), of NPs in the tumor vicinity, a phenomenon known as the Enhanced Permeability and Retention (EPR) effect. Although there is still controversy regarding the lack of uniformity in the observed EPR effect between species, at least in some human tumors the passive targeting of macromolecules and NPs has been demonstrated (Bjö et al., 2017).

On the other hand, a large number of genes (including many cell surface and nuclear receptors genes) are amplified or overexpressed in cancer cells. With the right surface ligands, NPs may be directed (i.e., actively targeted) to specifically bound those receptors (Shi et al., 2017). Among the 155 articles of SLN applicable to cancer, 23 of them involved some active targeting strategy. In contrast to the wide variety of payloads mentioned above, the targeting moieties belong, in the majority of cases, to one of two main classes: peptides and proteins (including antibodies, 70%) or folate residues (26%).

The α-isoform of the folate receptor, which is normally expressed at the apical surface of epithelial tissues and overexpressed in tumor cells of epithelial origin. Hence, it could be used to promote drug uptake by cancer cells via receptor-mediated endocytosis, by attaching folate residues to a nanoparticle surface (Holm and Hansen, 2020). Indeed, this strategy was successfully applied to the design and preparation of folate-grafted SLN loaded with irinotecan (Rajpoot and Jain, 2020) and a combination of resveratrol and ferulic acid (Senthil Kumar et al., 2020) for the treatment of colorectal cancer. Another example is found in integrin αvβ3, an adhesion molecule presented in all cells but overexpressed in several types of tumors. It has been demonstrated that its interaction with the RGD tripeptide (arginine-glycine-aspartic) leads to a number of cell functions that ultimately contributes to angiogenesis and metastasis (Martínez-Jothar et al., 2020). Conjugation of SLN with RGD increased *in vitro* antitumor efficacy and *in vivo* cytotoxicity in comparison with non-targeted SLN (Zheng et al., 2019).

On the other hand, targeting ligands may be intended to promote the passage through physiological barriers like the blood brain barrier (BBB), to reach a site of action at CNS. This approach was applied in the formulation of docetaxel-loaded SLN functionalized with angiopep-2 (A-SLN), that specially binds to the low-density lipoprotein receptor related protein 1 (LRP1) overexpressed at the BBB. Higher *in vitro* cytotoxicity and BBB permeability were found for A-SLN, attributable to receptor-mediated endocytic processes (Kadari et al., 2018). Moreover, dual-approaches or combinations are also possible: Kuo and Lee (2016) achieved an increased toxicity on tumor cells by incorporating two antibodies for a two-stage targeting: first to BBB cells (83-14 MAb), and then to glioblastoma cells (AEGFR).

It is worth highlighting that, in order to efficiently conjugate the targeting moiety to the SLN, much more complicated synthesis methods are required. The systems are no longer made of the simple mix lipid/s - surfactant/s - drug/s. Instead, other reagents, solvents and reaction steps must be incorporated to the preparation protocol.

There are several options for attaching a targeting ligand to an SLN, such as linking a fatty acid of the NP with an amino group of the ligand (Siddhartha et al., 2018), an amino group of a phospholipid to an acid group of the ligand (Rajpoot and Jain, 2020), or an amino group of the chitosan coating with an acid group of the ligand (Senthil Kumar et al., 2020), among others. Regardless of the particulars, these examples end in the formation of an amide, which is by far the most widely used bond to attach ligands to the surface of lipid nanoparticles. In order to efficiently form an amide bond, activating reagents are required (carbodiimide, H-hydroxysuccinimide, etc.), and several steps must be performed in potentially toxic organic solvents. Therefore, the improvement in efficacy and/or biodistribution aimed by means of active targeting strategies is achieved by sacrificing what is (possibly) the main advantage of SLN/NLC: their green synthesis and safety profile.

Perhaps a better option is to use other types of chemical bonds instead of covalent bonds. Souto et al. (2019) synthesized extremely positive SLNs (ca. +70 mV, by choosing cetyltrimethylammonium bromide as surfactant) able to electrostatically interact with negatively charged streptavidin (pI = 5). The objective was to bind a biotinylated antibody (CAB51, against human epithelial growth receptor 2, HER2), taking advantage of the strong interaction between streptavidin and biotin. The goal was somehow accomplished, since *in vitro* assays revealed an improved internalization of the targeted NPs on a HER2 positive cell line (BT-474) compared to a HER2 negative cell line (MCF-7). But further optimization will be necessary to reduce the cytotoxicity exhibited by the nanoparticles themselves, which according to the authors was probably due to the cationic surfactant and/or their positive charge (Souto et al., 2019).

Last, but not least, the economic aspect must be mentioned. The costs of taking a novel nanomedicine into the clinic can be a significant obstacle for the introduction of new nanomedicines in the pharmaceutical market (Hare et al., 2017). Histories of success like Abraxane, with sales of nearly $1 billion by 2015 (van der Meel et al., 2017), and efforts like the Cancer Moonshot Task Force recommendation to enhance public–private partnerships (Jaffee et al., 2017) are expected to encourage drug developers to invest time and resources for cancer R&D.

Another area that could take much advantage from pharmaceutical nanovehicles is the one related to antibacterials, antivirals, antiparasitic and antifungals, grouped as **antimicrobials** in Figure 2.

All the reviewed articles corresponding to SLN/NLC applications to antiviral therapies present as main advantage the optimization of the distribution / accumulation of the drug in the site of action, as well as an improved biodistribution and diminished cytotoxicity.

As we will discuss later, drugs whose site of action is at the CNS level always represent a challenge in terms of biodistribution in order to achieve effective concentrations in the brain. Lipid NPs of zidovudine and saquinavir intended for the CNS showed promising results in cell cultures *in vitro* (Kuo and Wang, 2014; Joshy et al., 2016), and SLN-based formulations of efavirez (Gupta et al., 2017) and nevirapine (Lahkar and Kumar Das, 2018) exhibited an improved central *in vivo* bioavailability (BA). In the case of efavirenz, the strategy was to circumvent the BBB by means of the nasal administration of the nanoparticles, while in the nevirapine case the administration was by intravenous (IV) route and the improved biodistribution was attributed to the coating (polysorbate 80), able to enrich the protein crown in ApoE, resulting in a higher passage through the BBB due to the contribution of receptor-mediated transcytosis (Li et al., 2018; Krishna et al., 2019).

Regarding antiviral formulations intended for systemic effect after oral administration (Gaur et al., 2014; Shi et al., 2015; Ravindra Babu et al., 2019), an interesting work by Ravi et al. (2014) evaluated the comparative performance of the protease inhibitor lopinavir (LPV)-SLN with respect to LPV alone and the combination of LPV-Ritonavir (RTV). LPV is co-formulated with subtherapeutic doses of Ritonavir to overcome its poor oral BA due to CYP3A4 metabolism and P-glycoprotein (P-gp) efflux, both inhibited by RTV. The LPV-SLN presented greater oral BA than the LPV-RTV combination, and in vitro metabolic stability and rat everted gut sac studies allowed the authors to conclude that the observed results were due to a combination of a metabolic protection and increased intestinal permeability of the drug encapsulated into the SLN (Ravi et al., 2014).

A very promising aspect, although not still fully addressed, of the use of lipid-based NP to the delivery of antibiotics is the possibility to overcome some of the drug resistance mechanisms acquired by bacteria. Multiple-drug resistance (MDR) may be acquired by either a mutation or the acquisition of new genetic material from an exogenous source, that results in a mutated version of a drug target, membrane protein, transporters or enzymes, as beta-lactamases. In the same manner as NPs may help to optimize the pharmacokinetic (PK) profile of a drug by reducing its metabolism and/or efflux by ABC transporters in humans, it is feasible to apply the same concept to overcome the resistance produced by similar mechanisms in bacteria (Christaki et al., 2020). The possibility to deliver biotechnological drugs encapsulated into SLN/NLC may also help to overcome MDR by exploring new therapeutic strategies, like interfering with the bacterial transcription process through the delivery of DNA molecules complexed with lipid NP (González-Paredes et al., 2019).

Moreover, lipid-based nanosystems offer several indirect-ways to address drug-resistance issues, by one or more of the following strategies:

• Achieving a sustained release profile of the drug, to maintain steady concentrations within its therapeutic concentration, and thus avoiding suboptimal levels which can promote resistant microbes selection (Nafee et al., 2014; Chetoni et al., 2016).
• Lowering the drug toxicity by encapsulation, allowing higher doses and/or treatment periods (Severino et al., 2015; Chaves et al., 2018).
• Increasing systemic BA (Chetoni et al., 2016; Banerjee et al., 2020) and CNS levels (Abdel Hady et al., 2020).
• Allowing pulmonary administration, with less unspecific distribution (Nafee et al., 2014; Gaspar et al., 2016, 2017; Maretti et al., 2017; Vieira et al., 2018).
• Promoting accumulation in target cells by means of active targeting (Maretti et al., 2017; Costa et al., 2018; Vieira et al., 2018; Hosseini et al., 2019; Banerjee et al., 2020).
• Increasing inhibitory effect (i.e., decreasing MIC) over bacterial strains (Severino et al., 2015; Pignatello et al., 2017; Ghaderkhani et al., 2019; Rodenak-Kladniew et al., 2019).

On the other hand, the very lipophilic groups of antiparasitic and azole antifungal agents highlight another advantageous aspect of lipid-based NPs. Due to its lipid components, SLN/NLC are able to solubilize highly lipophilic (i.e., aqueous insoluble) drugs, and keep them in a stable suspension, avoiding the use of large amounts of surface-active compounds and improving the biopharmaceutical performance after oral (Souza et al., 2014; Aljaeid and Hosny, 2016; Omwoyo et al., 2016; Rehman et al., 2018), ocular (Mohanty et al., 2015; Kumar and Sinha, 2016), and/or parenteral administration (Ahmadnia et al., 2013; Permana et al., 2019).

The hydrophobic constituents of lipid-based nanosystems provide a suitable environment for the entrapment of hydrophobic drugs, positioning SLN/NLC as a promising tool, particularly relevant in the current context where there is a growing trend toward more lipophilic drug candidates. *In silico* drug discovery strategies, high throughput screening methodologies and the more classical lead-optimization programs tend to favor compounds with higher pharmacological potency, in detriment of other properties that may be desirable from a physicochemical or pharmacokinetic point of view. On the contrary, it is a known fact that when the biopharmaceutical characteristics of drug candidates are addressed in early stages of discovery programs the consequence is an increase in failures due to lack of efficacy and, to a lesser extent, for toxicity concerns (Kola and Landis, 2004). Therefore, pharmaceutical chemists will always have to deal with the PK/PD & toxicity balance, and the aqueous solubility will remain to be a critical factor in drug discovery. Proof of this is that, to date, approximately 39% of the marketed drugs (Benet, 2013) and 60% of the new chemical entities (Kovačević, 2020) belong to the biopharmaceutical categories that group *low solubility* drugs (i.e., BCS classes 2 and 4).

## Routes of Administration Proposed for SLN /NLC

**FIGURE 3:**
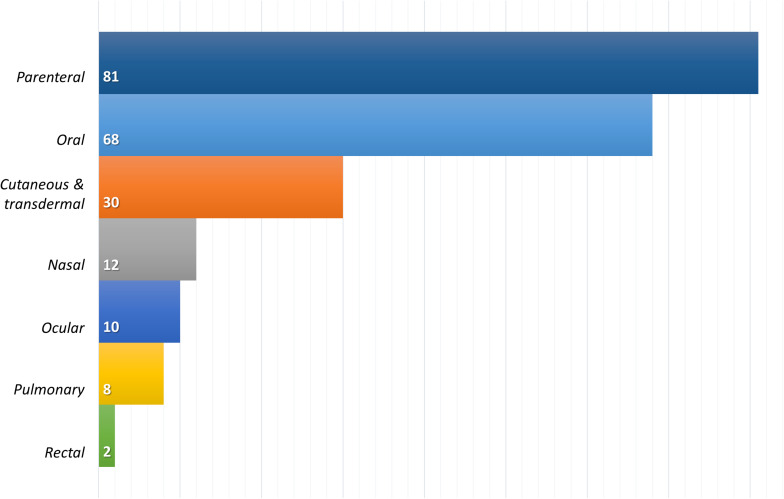
Distribution of the 2013–2020 reviewed publications on SLN/NLC, by the proposed route of administration. Only publications with a PD and/or PK studies were considered (211 of 371).

As can be seen in Figure 3, the most commonly proposed administration route for lipid-based nanosystems is the parenteral route, closely followed by the oral route. Both administration routes seek to achieve systemic effects of the encapsulated drugs, but the trend described is opposite to the current distribution of pharmaceutical products in the market, where the oral route of administration is the preferred and most widely used route for drug administration.

Parenteral routes, on the other hand, allow the delivery of drugs directly to the systemic circulation with no absorptive barriers to overcome or with minimal restrictions, as in the case of the intramuscular and/or subcutaneous route. More than 50% of the lipid nanosystems assayed by parenteral routes (46 out of 81) corresponds to anticancer drugs, a therapeutic field where IV route remains predominant, in spite of its non-negligible negative aspects, such as invasiveness, associated risks, inability to self-manage and higher technological requirements to be manufactured with suitable microbiological quality (Ruiz and Scioli Montoto, 2018).

### Oral Route

The oral route, being a natural route of entry of substances to the organism, enjoys the greatest acceptability, as well as some technological advantages, since oral pharmaceuticals mostly comprise non-sterile solids dosage forms. For a successful therapy by the oral route, though, a drug must generally fall within certain ranges of lipophilicity, molecular weight, and hydrogen bonding ability, as well as aqueous solubility and permeability, which altogether contribute to its *druglikeness* (Di and Kerns, 2016).

Curcumin, for example, represents a real challenge for its formulation as oral product, due to its very low aqueous solubility, poor absorption, rapid metabolism and pH-dependent degradation rate (Sanidad et al., 2019). Oral BA of curcumin has been reported to be as low as 1% (Ma et al., 2019). On the other hand, successful outcomes of curcumin in both preclinical and clinical trial of different diseases make it a very promising drug, that seems to be able to modulate several cell signaling pathways and, thus, holds a great therapeutic potential against a wide range of human diseases (e.g., cancer, infections, inflammatory, metabolic and neurodegenerative diseases, among others) (Gupta et al., 2013). Furthermore, there is enough evidence to support the hypothesis of dose-dependent pharmacological activity of curcumin, with the anticancer properties corresponding to the highest doses (Doktorovova et al., 2018).

Regardless the somehow inconsistent reports on curcumin oral BA (possible due to variations in experimental conditions), there is agreement on the positive increment in the oral BA of curcumin formulated within nanosystems, with respect to the free drug in solution (Anand et al., 2007). Predictably, the publications reviewed here confirmed that trend, since curcumin oral BA achieved with SLN/NLC was from 2 to more than 10-fold higher than that of the free drug solution (Kakkar et al., 2013; Ramalingam and Ko, 2015; Baek and Cho, 2017). The examination of the PK profiles seems to indicate that the BA improvement of curcumin is related to the combined effect of a higher absorption and a minor elimination of the encapsulated drug, similarly to what was described for LPV-SLN.

Among the reviewed articles, the aforementioned trend is confirmed by many other examples. Administration as SLN/NLC greatly increases the oral BA of drugs with very low aqueous solubility such as aripiprazole (Silki and Sinha, 2018), rhein (Feng et al., 2017), zaleplon (Dudhipala and Janga, 2017), miconazole (Aljaeid and Hosny, 2016), raloxifene (Singh et al., 2013; Tran et al., 2014), efavirenz (Gaur et al., 2014), doxorubicin (Yuan et al., 2013), asenapine (Patel et al., 2019b), linagliptin (Veni and Gupta, 2020), and niclosamide (Rehman et al., 2018), among several others.

The group of calcium channel blockers derived from dihydropyridine, for example, is characterized by its low oral BA due to its low water solubility and high rate of first-pass metabolism. Administered as lipid-based nanosystems, significant increases in the oral relative BA was observed for isradipine [4.5-fold, (Kumar et al., 2018)], nisolpine [2.5-fold, (Dudhipala et al., 2018)], felodipine [3.2-fold, (He et al., 2020)], and cilnidipine [2.4-folds, (Diwan et al., 2020)]. These are very promising results taking into consideration that, when administered as conventional formulations, the oral BA of these four drugs is in the range of 5-20% (Wishart et al., 2018).

These previous examples illustrate the possibilities and advantages offered by lipid NPs for oral pharmacological therapy. Nonetheless, despite the abundance of PK and pharmacological “advantages,” the underlying mechanisms are not yet fully understood. Regarding the higher oral BA, evidences suggest a combination of four possible effects:

(1) Drug protection against both chemical and enzymatic degradation. Encapsulation in a nano-sized lipid matrix may reduce or retard a drug pH-dependent hydrolytic degradation (Baek and Cho, 2017), as well as the drug inactivation by the gastrointestinal (GI) tract digestive enzymes, which may be crucial for the oral administration of biological drugs. It has been demonstrated, for example, that whereas free salmon calcitonin was almost completely degraded *in vitro* by pancreatin in 15 min, the drug encapsulated into SLN exhibited a much slower degradation kinetics, and was still detectable in the reaction media up to 12 h (Fan et al., 2014).
(2) Lipid effect on solubility improvement that allows higher effective doses. Shangguan et al. (2015) evaluated the BA of silymarin in Beagle dogs, comparing the administration as intact drug-loaded SLN/NLC and as a lipolysate produced by the enzymatic action of pancreatic lipase over the lipid NP. The lower BA obtained with the lipolysate was in agreement with the loss of drug in the formulation, since the micelles formed in the GI to facilitate the uptake of lipophilic compounds (known as “mixed micelles,” and mainly composed by phospholipids, bile salts, and cholesterol, Yao et al., 2017) cannot keep all silymarin in suspension, and drug precipitation occurs. In other words, when the BA values are corrected by a factor that accounts for the true dose administered (i.e., amount of drug remaining in suspension), it may be concluded that the lipolysis pathway is the predominant mechanism underlying the enhanced oral BA of a drug formulated as lipid NPs, whereas the absorption of intact NPs only plays a minor role.
(3) Major retention in the GI tract. When a lipid-based NP reaches the GI tract, its hydrophobic surface tends to adhere to the mucus layer, whose superficial layers are quickly and continuously cleared as protection against particles and pathogens (Maisel et al., 2015). To minimize such effect, “mucus penetrating particles” (MPP) can be formulated. MPP have a smaller size than the mucus layer, and a hydrophilic, non-muco-adhesive surface (generally obtained with PEG cover) (Schneider et al., 2017). In spite of their lipid nature, these particles are capable of getting in contact with the GI epithelium, thus achieving prolonged absorption of the encapsulated drug (Yuan et al., 2013).
(4) Finally, in the same way that NPs protect the drug from degradation by enzymes present in GI lumen, they can also prevent/reduce the degradation by metabolic enzymes, as in the lopinavir example mentioned in the previous section (Ravi et al., 2014). Reduction in pre-systemic *in vivo* metabolism may occur due to less hepatic metabolism (e.g., NP accessing portal circulation as such, see the next section) and/or increased lymphatic uptake of the NPs by the lymphatic vessels in the gut (Baek and Cho, 2017; Bernier-Latmani and Petrova, 2017). Working with a chylomicron production blocking agent, Patel et al. found that the lymphatic uptake represented nearly 30% of the drug oral BA (asenapine maleate, administered as a SLN suspension) (Patel et al., 2019b).

Additionally, we can mention one more effect, common to all orally administrable pharmaceutical forms: the presence of excipients that may affect the rate and extent of drug absorption (FDA/CDER, 2015). Tensioactives and surfactants belong to this group and are usually present at high concentrations in SLN/NLC.

**FIGURE 4:**
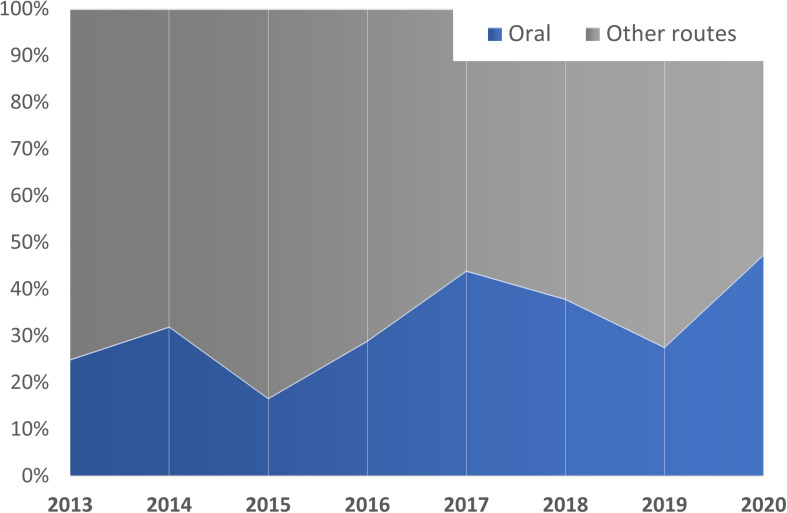
Time-trend of the SLN/NLC intended for oral administration. Only publications with *in vivo* (PD and/or PK) studies are considered (211 of 371).

It is likely that the combination or synergistic action of all these effects is the cause of the large increase in oral BA associated with lipid nanovehicles and, in turn, of the increasing trend in the selection of this route of administration for SLN/NLC (see Figure 4). Furthermore, due to all the previously described effects, lipid-based NP have been examined for the oral delivery of peptide therapeutics, such as salmon calcitonin (Chen et al., 2013; Fan et al., 2014) and insulin (Hecq et al., 2016; Xu et al., 2018; Alsulays et al., 2019).

### Percutaneous Route

According to the FDA classification, the percutaneous route of administration consists in the administration of drugs through the skin (FDA, 2017), and it comprises two groups of pharmaceutical products: those intended to exert a local action, at some level of the skin, and those that seek a systemic action of the drug, also known as transdermal formulations. Of the 30 reviewed publications corresponding to preparations to be administered onto the skin, only 3 of them (10%) seek systemic action of the drug: metformin for diabetes (Sharma et al., 2013), avanafil for erectile dysfunction (Kurakula et al., 2016) and piperine for rheumatic arthritis (Bhalekar et al., 2017), while the remaining 90% consists of formulations for local action.

The skin is composed of two main histological layers, the epidermis at the surface, and the dermis below. In turn, the outermost layer of the epidermis is the stratum corneum or the *horny layer*, which is the real barrier that prevents the entry of foreign (and potentially harmful) substances into the body. The stratum corneum is formed by cells named corneocytes or keratinized cells, surrounded by shallow valleys that comprise the intercellular regions filled with lipid multilamellae, rich in ceramides, fatty acids and cholesterol.

The ability of a drug to penetrate the skin depends on its physicochemical properties (mainly its size, molecular weight, pKa and partition coefficient) as well as the vehicle in which it is formulated. There are substances known as “permeation enhancers” which are capable of reversibly disorganizing the stratum corneum, facilitating the drug entry (e.g., fatty acids and alcohols with long carbon chains, surfactants, terpenes and fatty esters, Gupta et al., 2019). These excipients are commonly used in classic semi-solids preparations like emulsions, lotions and ointments, as well as of their more recent relatives’ lipid-based nanoformulations. Hence, it is logical that these formulations are, among other nanosystems, the first choice for percutaneous/transdermal applications.

The most studied nanoparticulate systems for percutaneous application are, by far, liposomes. And although it has been shown that the constituent lipids of liposomes are capable of reaching the deeper layers of the skin (i.e., the dermis), it still remains unclear if they can act as carriers, penetrating through the skin, or if they only act as penetration enhancers, changing the skin physical properties in a way that facilitates the (free) drug penetration through it (Peralta et al., 2018).

Nevertheless, pharmaceutical formulations of SLN/NLC have proven to be useful for percutaneous administration of drugs, to treat diseases or alterations at every level of the skin: the epidermis, like fungal infections (Vaghasiya et al., 2013), hyperpigmentation (Ghanbarzadeh et al., 2015), skin cancer (Taveira et al., 2014; Geetha et al., 2015; Khallaf et al., 2016; Tupal et al., 2016) and atopic dermatitis (Kang et al., 2019); the dermis, as in the case of anti-inflammatory drugs (Dasgupta et al., 2013; Gaur et al., 2013; Raj et al., 2016; Daneshmand et al., 2018; Shinde et al., 2019) and local anesthetics (You et al., 2017); both dermis and epidermis, like psoriasis (Sonawane et al., 2014) and infections by herpes virus (Gide et al., 2013; El-Assal, 2017) and; the appendices, like hair follicles (Hamishehkar et al., 2016).

## Disposition in the Body and Penetration to the CNS

Studying the distribution of these drug delivery nanosystems within the body is one of the main research challenges in the field. Although the advances that so far have been achieved in terms of the development of SLN/NLC are relevant, only a few works are dedicated to a detailed study of the fate of this type of NPs once they enter the organism. This section is intended to describe the main mechanisms involved in the uptake, transport and distribution of NPs into the body, as well as how these structures face the natural barrier that protects the CNS.

### Gastrointestinal Absorption of Lipid Nanoparticles

As mentioned in previous sections, SLN and NLC proved to be particularly promising for the enhancement of drugs oral BA, by avoiding their degradation in the GI tract, improving their solubility and dissolution rate, increasing their contact with the epithelium and/or minimizing their efflux by P-gp and other drug transporters. Either by one or by several of these mechanisms, orally administered lipid NPs can effectively increase the area under the plasmatic concentration curve of the encapsulated drug, as described by the curcumin examples. In the same manner, Wang and co-workers managed to improve the oral BA of [6]-shogaol, an alkylphenol extracted from ginger roots, of great interest for its antitumor, antioxidative and antirheumatic properties, as demonstrated by the greater AUC exhibited by the drug incorporated into SLN, but also by a significant decrease of serum uric acid, IL-1β and TNF-α levels with respect to free [6]-shogaol tests (Wang et al., 2018).

NPs constituents could have an effect on the intestinal absorption enhancement: lipids are able to increase the intestinal mucosa permeability (Talegaonkar and Bhattacharyya, 2019) and, although some controversy exists (Ball et al., 2018), modulation of the tight junctions (Beguin et al., 2013). On the other hand, tensioactives, surfactants and hydrophilic coatings like chitosan have also been proposed to enhance BA by the opening of tight junctions (Han et al., 2019; McCartney et al., 2019).

Overall, when it comes to the demonstration of the GI absorption of intact NPs, evidence is much scarcer. Regarding cellular uptake of the NPs, endocytosis is considered the predominant pathway (Wang et al., 2011; Patel et al., 2019b). There are two principal endocytosis mechanisms: pinocytosis and phagocytosis. Cellular uptake by macrophages (phagocytic cells) is reserved for those particles larger than 0.5-10 μm (Zhao et al., 2011). Pinocytosis, on the other hand, occurs in all types of cells and is responsible of the uptake of smaller particles (50 nm–5 μm). It may be further classified into clathrin-dependent (or clathrin-mediated endocytosis, CME) and clathrin-independent, the latter comprising caveolae-mediated and clathrin/caveolae-independent endocytosis, and macropinocytosis (also clathrin/caveolae independent, but for the internalization of larger particles, similar to phagocytosis) (Sahay et al., 2010).

This classification is based on the proteins (*clathrin* and *caveolin*) involved in the endocytic process, and thus it may overlap with other classifications based on different criteria, like *receptor mediated* or *adsorptive* endocytosis. For example, it was proposed that folate grafted NPs may be internalized by clathrin-mediated, clathrin/caveolae-independent (Sahay et al., 2010) and/or caveolae−mediated endocytosis (Wang et al., 2011). Rajpoot and Jain (2018, 2019) employed folic acid (FA) as targeting ligand of SLN containing oxaliplatin and irinotecan for the treatment of colorectal cancer, finding a slightly higher uptake (and higher toxicity) of the FA-SLN compared with the non-targeted SLN in HT 29 cells.

Cellular uptake via the LDL receptor, on the other hand, occurs by CME (Sahay et al., 2010; Wang et al., 2011). This pathway has been explored for the active targeting of rosuvastatin loaded SLN (Beg et al., 2017). To mimic the outer layer of LDL particles, rosuvastatin-SLN were coated with phospholipids (phospholipon 90G and/or PEGylated DSPE), and the endocytosis process was studied in Caco-2 cells by using filipin and sucrose as specific blockers of caveolae and clathrin-mediated endocytosis, respectively. A significant reduction in the cellular uptake of the drug in the presence of sucrose was found, providing indirect evidence of the lipid NPs internalization via the LDL receptor by CME (Beg et al., 2017). CME was also the predominant pathway responsible for the internalization of stearic acid based-SLN in human epithelial cells (lung A549 and cervical HeLa cells) (Shah et al., 2016b).

It is worth mentioning, however, that the successful endocytosis of a NP does not guarantee its absorption: once in the intracellular space of an epithelial cell, the NP should be further exocytosed on the basolateral side to reach the capillary vessels, in a process known as transcytosis. A comprehensive work by Chai et al. (2014) showed that SLN (60-100 nm) with no targeting ligand were internalized mostly by caveolae and clathrin-mediated endocytosis in MDCK cells. Once inside the cells, lysosomes were the main destination of the endocytic vesicles, whereas the transcytosis to the basolateral side account for only about 2.5% of the total NPs (Chai et al., 2014). This result is in line with those of Hu et al. (2016), who concluded that orally administered SLN exhibit significant cellular uptake but fail to penetrate cell monolayers. The authors studied the *in vivo* distribution of SLN and their interaction with biomembranes by water-quenching fluorescence, and could not find evidence of penetration of integral nanocarriers (Hu et al., 2016).

In a follow-up article, however, the same authors found some evidence of intact uptake of the SLN from the GI lumen to the circulation, apparently through the lymphatic route, but representing only a minor contribution to the oral BA of a drug (Ma et al., 2017). Regarding the lymphatic uptake, lipid NPs may access the lymphatic system through the intestinal lipid transport system (O’Driscoll, 2002) as well as by *transcellular* passage, by the association with chylomicrons after the digestion of the lipid nanosystems, and by *specific passage* through the M-cells in the Peyer’s patches (Salah et al., 2020). In the last case, NPs size is a relevant variable, since particles larger than 100 nm will be retained longer in the Peyer’s patches, while smaller ones will be transported to the thoracic duct (Bummer, 2004). Surface charge is also a key feature that affects this process, with anionic particles being more rapidly absorbed by the lymphatic route (Yu et al., 2019).

### Systemic Circulation and Protein Corona Formation

Due to their small size and, thus, their large surface area, NPs are characterized by a high free energy. Accordingly, the interaction with different macromolecules, when they are in contact with biological fluids, will be favored. Once NPs have reached systemic circulation, another inconvenience is presented: a biological macromolecules-cover known as *protein corona* (PC) begins to form upon their surface.

This corona is composed of two layers formed in a time dependent manner. During a first stage, a loose layer named *soft corona* starts to settle. This corona is composed by low-affinity proteins with a high relative abundancy, which are in constant exchange with the biological medium and NPs surface, in a process known as “Vroman effect” (Vroman, 1962). Then, in a second stage, low-affinity proteins begin to be replaced by those with lower relative abundance, but with a higher surface affinity, staying close to it for a longer period. Is in this stage where the formation of the *hard corona* is evidenced (Baimanov et al., 2019). It follows that the “chemical identity” of the NP is not equal to its “biological identity:” the formation of the PC (both soft and hard) substantially changes the nanosystems properties, being able to impact in their size, shape, and final surface composition (Lima et al., 2020), turning them into a new biological identity.

Gessner et al. (2002) studied the influence of surface charge density on protein adsorption on polymeric NPs, concluding that the higher the surface charge density, the higher the amount of proteins adsorbed. The authors observed no qualitative change in the pattern of adsorbed proteins.

As expected in a complex biological process, the pattern of protein adsorption does not only depend on the protein capacity to access the particles surface, but also on the characteristics of the surface itself (Göppert and Müller, 2003). As it was previously described for polysorbate 80 coatings, the use of different Poloxamer in the formulation of SLN facilitates the adsorption of different proteins *in vitro*: the MW Poloxamer 184 and Poloxamer 235 showed a high ApoE absorption, which mediates the uptake through the BBB. Even more interesting, these lipid NPs showed a high adsorption of ApoA-IV (involved in the promotion of brain uptake) and a low adsorption of ApoC-II (responsible for the inhibition of receptor mediated binding and uptake of lipoproteins) (Goppert and Muller, 2005).

During the formation of the PC, the incorporation of proteins of the complement system also known as *opsonins* occur. The complement system is part of the innate immune system and facilitates the recognition of NPs by the mononuclear phagocytic system (MPS), which in turn leads to an increase of NPs clearance and a reduction of their systemic residence time.

A study by Fang et al. (2006) confirmed the dependency of phagocytosis by murine macrophages with the particle size of the NPs, as well as with the molecular weight of methoxy polyethylene glycol (MePEG) used for coating. The authors observed that those NPs coated with MePEG of the same molecular weight, showed a higher distribution half- life as the size decreased. On the other hand, the uptake by macrophages was decreased by increasing the coating molecular weight (Fang et al., 2006).

Previously, Müller et al. (1996a) had studied the dependence of the uptake by macrophages with hydrophilicity and steric hindrance given by different types of emulsifiers (e.g., poloxamine 908 and poloxamer 407), demonstrating that an increase in hydrophilicity and steric hindrance diminished the uptake by macrophages. Xiao et al. (2011) worked with different types of D-aspartic acids and D-lysines-derivatized telodendrimers which possessed different surface charges. Those dendrimers composed by D-aspartic acids (negatively charged) and the acetylated derivatized NPs (neutral charge) showed a lower macrophage uptake in comparison with the cationic D-lysines (positively charged) (Xiao et al., 2011).

To achieve distribution beyond the liver, NPs need to avoid rapid opsonization and clearance by the MPS (Müller et al., 1996a). A great deal of work has been devoted to developing the so-called stealth NPs, which are “invisible” to macrophages (Brigger et al., 2012; Rudhrabatla et al., 2019, 2020), due to the PEG chains on their surface (*PEGylated* NPs) (Hadjesfandiari, 2018). This coating prevents or delays the formation of the PC and, thus, NPs exhibit a prolonged half-life in the blood compartment (Pelaz et al., 2015). However, a number of limitations to the use of PEG have also been described, such as the production of anti-PEG antibodies or the impairment of cellular internalization by the stealth coating (Baimanov et al., 2019). Depending on the nature of the nanovehicle, different approaches have been explored to circumvent these limitations, e.g., stimuli-responsive PEG-derivatized nanocarriers (Fang et al., 2017).

### Passage Through the Blood Brain Barrier (BBB)

The BBB is a semipermeable structure composed mainly of the microvasculature of the CNS. This barrier is formed by a continuous layer of endothelial cells integrated to a complex systems that regulates the bloodstream-to-CNS movement of molecules, ions and cells, also responsible for the homeostasis regulation (Ayloo and Gu, 2019). Unlike the peripheral endothelium, BBB endothelial cells present a high content of mitochondria, lack of fenestrations and pinocytic activity and, as a salient characteristic, particularly occlusive tight junctions formed by several transmembrane proteins (such as claudins, occludins, and junctional adhesion molecules or JAM, among others Stamatovic et al., 2008), that efficiently limit the paracellular diffusion pathway. Another characteristic of the BBB is the expression of efflux transporters of the ABC (ATP-binding cassette) superfamily, transmembrane proteins responsible for pumping xenobiotics or toxic substrates out of the intracellular space, avoiding their access to the CNS since they are localized almost exclusively at the luminal membrane of the endothelial cells (Pardridge, 2020). These transporters are one of the main causes of multi-drug resistance phenomena, which is why they are also known as multidrug resistance (MDR) proteins (the most representative one being P-gp). It has been proposed that encapsulating a drug into a NP may help to bypass these transporters (Cavaco et al., 2017; Sadegh Malvajerd et al., 2019).

The transport of substances through the BBB may occur by four main mechanisms (Xie et al., 2019)*: paracellular diffusion*, reserved for small water soluble substances; *transcellular diffusion*, which is more relevant for molecules with an appreciable lipophilicity and a molecular weight smaller than 450 kDa; *carrier-facilitated diffusion and active transport*, responsible for the passage of specific molecules like small peptides, sugars, monocarboxylic acids, amino acids, organic anions and cations, neurotransmitters and nucleosides; and *endocytosis*, this pathway has been reported for the passage of peptides and proteins through the BBB, such as insulin and the insulin-like growing factor (IGF-I and IGF-II) (Patel et al., 2013).

Although it has been suggested that NPs can enter the CNS by the paracellular pathway (through the transient opening of the tight junctions, as in the case of chitosan-coated NPs, Yu et al., 2013; Zhang et al., 2014), there is now evidence suggesting that the predominant mechanism is the NPs endocytosis. Once inside the endothelial cell, NPs can be exocytosed to the other side (*transcytocis* of the NP) or released in the intracellular space, promoting their access to the CNS (Saraiva et al., 2016).

Since endocytosis of NPs by the BBB endothelial cells seems to be predominantly mediated by receptors, many efforts have been made with SLN surface functionalization to enhance their CNS availability (Ceña and Játiva, 2018; Kuo et al., 2019; Wang et al., 2019), as discussed in Section 2 for the functionalized angiopep-2 NPs to treat glioblastoma multiforme (Kadari et al., 2018).

As another example, functionalized ApoE NPs appear to be particularly promising in such way. ApoE possess high affinity receptors along the BBB, a characteristic that has been exploited for the delivery of drugs in functionalized nanosystems with this protein (Zensi et al., 2009). In a series of studies, Neves *et al.* investigated the cellular uptake of ApoE-grafted SLN by hCMEC/D3 cells, as a model of human BBB, and found that functionalized NPs were better internalized than non-functionalized ones, due to the specific recognition of the targeting ligand by the highly expressed LDL receptors (Neves et al., 2015, 2016, 2017). Moreover, by the use of specific inhibitors, CME was identified as the preferential endocytic pathway for ApoE-SLN (Neves et al., 2017).

## Clearance Mechanisms and Toxicological Aspects

Achieving nanocarriers with low or no toxicity for the organism and the environment is one of the biggest challenges in designing drug delivery nanosystems. Ideally, the drug carrier should be rapidly removed from the body after the drug has been released. Lipid-based NPs sizes are far over the renal filtration threshold (Yang et al., 2019), for what, once in the bloodstream, they have to be opsonized by serum proteins and subsequently uptaken by the MPS in specialized organs (i.e., liver, kidney, spleen, lungs, and lymph nodes) for their efficient elimination from the body (Di Ianni et al., 2017). Despite the fact that fenestrations in the spleen may filter out particles larger than 200 nm, particle deformability can allow large particles to squeeze through them and remain in the bloodstream (Park et al., 2017).

**FIGURE 5:**
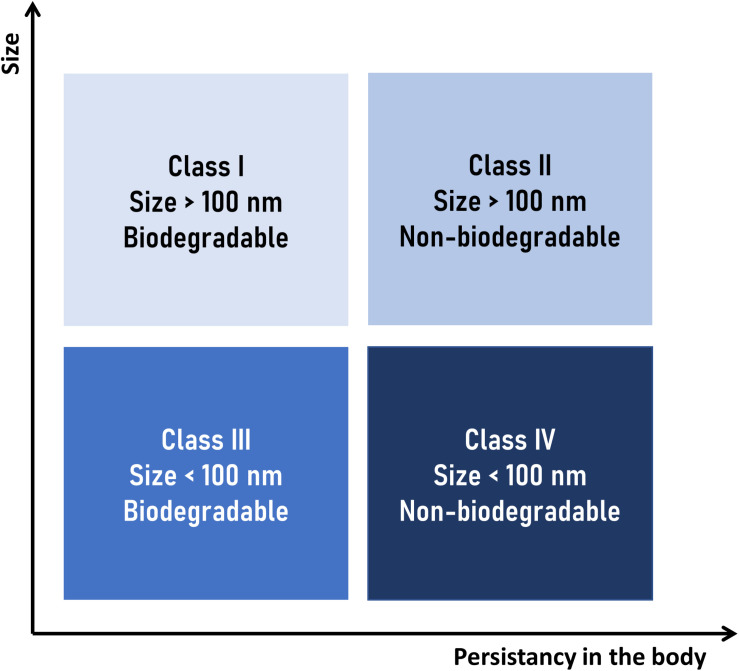
Nanotoxicological classification system (NCS) suggested by Keck and Müller (2013). NPs are placed in one of four categories according to their size and biodegradability: class I (no or low risk), classes II and III (medium risk), and class IV (high risk).

Considering the clearance mechanism described above, Keck and Müller (2013), a “nanotoxicological classification system” (NCS), as a rational approach to assess the potential risks of toxicity of a given nanocarrier. According to that system, a nanocarrier is placed in one of four categories according to their size and biodegradability (Figure 5):

• Class I (no or low risk), for nanosystems of size above of 100 nm and made of biodegradable materials.
• Class II (medium risk), for nanosystems of size above 100 nm but made of non-biodegradable materials.
• Class III (medium risk), for nanosystems of size below 100 nm, made of biodegradable materials.
• Class IV (high risk), for nanosystems of size below 100 nm and made of non-biodegradable materials.

The size limit between classes of 100 nm was adopted considering the greater distribution in the organism of smaller particles (e.g., ease of access to the CNS), as well as the greater probability of non-specific endocytosis in off-target cells. However, when applying the NCS, it should be taken into consideration that larger particles can also be internalized by the cells through other mechanisms (Zhao et al., 2011; Danaei et al., 2018).

With regard to the biodegradability of the nanocarrier materials, SLN and NLC are generally considered as members of the NCS classes I or III, since they are composed of physiologically compatible lipids (fatty acids, glycerides or other fatty acid esters, sterols, sterol esters, waxes, etc.).

As stated before, a NP in the bloodstream will be taken up by the MPS. After the opsonization and phagocytosis has occurred, the resulting phagosome needs to “mature” to a phagolysosome [by a series of fusion and fission interactions with endosomes and lysosomes (Rosales and Uribe-Querol, 2017)]. The phagolysosome possesses a unique membrane composition to resist a very acidic and degradative environment, necessary to the final digestion of its content. Internalized non-biodegradable materials may however exert several cytotoxic effects, contributing to chronic inflammation and progressive tissue injury (Gordon, 2016; Azarnezhad et al., 2020).

It must be noted that toxicological outcomes are strongly dependent on the administration route: for instance, orally administered SLN/NLC can be eroded and degraded by bile salts and pancreatic lipase in the body (Müller et al., 1996b; Agrawal et al., 2014).

Although the previously described classification system may seem an excessively reductionist approach to the matter of nanotoxicology, it is a valuable tool in the current state of research of pharmaceutical nanovehicles. A huge number of different nanosystems are being proposed for drug delivery applications based on promising results in terms of their PK and/or PD performance, but for which the multiple aspects that could generate adverse events or toxicity in patients are still to be studied in detail.

One of the aspects not directly addressed by the NCS is the effect that the surface charge of the particles may exhibit on the toxicity or clearance mechanisms. In order to enhance cellular uptake, NPs are sometimes formulated with a positively charged surface, to facilitate electrostatic interaction with the negatively charged plasma membranes of cells, hence promoting internalization by non-selective, adsorptive mediated endocytosis (Ayloo and Gu, 2019). An example of this strategy is found in lipoplexes (a combination of negatively charged nucleic acids and positively charged lipids), which have demonstrated their ability to effectively deliver their load to target cells (Kowalski et al., 2019; Pardridge, 2020). Positively charged lipid NPs were also proposed for carrying nucleic acids (Kong et al., 2013; Fàbregas et al., 2014; Shi et al., 2014; Kotmakçı et al., 2017; Küçüktürkmen and Bozkır, 2018; González-Paredes et al., 2019). Despite these advantages, positively charged NPs have been associated with several toxic effects (Azarnezhad et al., 2020). Based on cell cultures experiments, some authors reported higher cytotoxicity values for cationic (vs. neutral and anionic) SLN (Karn-orachai et al., 2016), while others postulate that cell cultures are able to tolerate high concentrations of cationic SLN/NLC without appreciable toxicity (Doktorovová et al., 2016). These apparently inconsistent results may be explained by the fact that surface charge is not the only toxicity determinant of a NPs, and that other covariables (such as the chemical composition of the NP) may also be considered.

Nevertheless, caution should be taken when working with cationic SLN/NLC, as also some *in vivo* toxicity reports may be found. For example, Wu et al. (2018) demonstrated that SLN with different surface charges and PEG densities resulted toxic to platelets (and, to a lesser extent, to red blood cells), and that the toxic effects were dependent of the surface charge (the higher positive charge, the worst) and PEG densities (the lower, the worst).

## Translation Into the Clinic of Lipid (But Not Solid) Nanoparticles

At the time of writing this review, a search was made on the website www.clinicaltrials.gov, finding 13 relevant results corresponding to the keywords “lipid” and “nanoparticles” (see Table 1). However, of those studies, only one comprise what can be regarded as *classical* SLN: oxiconazole-loaded stearic acid NPs, further included in a carbopol gel formulation for the topical treatment of topical tinea infections (Mahmoud et al., 2020).

**TABLE 1:** Lipid Nanoparticle Drug Delivery Systems (LNDDS) on currently active clinical trials (terminated or withdrawn studies were excluded).

Track number	Status	Drug	Disease	Route of administration
***siRNA therapy***				
NCT01960348	Phase III	Patisiran (ALN-TTR02)	hTTR - mediated amyloidosis	IV infusion
NCT01858935	Phase I	ND-L02-s0201	Hepatic fibrosis	IV infusion
NCT02227459	Phase I	ND-L02-s0201	Hepatic fibrosis	IV infusion
NCT01437007	Phase I	TKM-080301	Primary liver carcinoma or metastatic liver cancer	Hepatic arterial infusion
***mRNA therapy***				
NCT04416126	Phase I	ARCT-810	OTC deficiency	IV infusion
NCT04442347	Phase I	ARCT-810	OTC deficiency	IV infusion
NCT03323398	Phase I Phase II	mRNA-2416	Solid tumors / Lymphoma / Ovarian Cancer	Intratumoural
NCT03739931	Phase I	mRNA-2752	Solid tumor malignancies / Lymphoma	Intratumoural
NCT04283461	Phase I	mRNA-1273	COVID-19	IM injection
***Others***				
NCT02971007	Phase II	CAMB	Vulvovaginal candidiasis	Oral
NCT02629419	Phase II	CAMB	Mucocutaneous candidiasis	Oral
NCT04148833	Phase II Phase III	Paclitaxel	Aortic and coronary atherosclerotic disease	IV injection
NCT03823040	Phase I	Oxiconazole	Tinea pedis / Tinea versicolor/Tinea circinate	SLNs loaded gel for topical application

*CAMB, encochleated amphotericin B; HSP47, Heat Shock Protein; IM, intramuscular; IV, intravenous; OTC, ornithine transcarbamylase; PLK1, polo-like kinase-1; TTR, Transthyretin.*

Two studies correspond to phase II trials of oral encochleated amphotericin B (CAMB). Cochleates are constituted by several layers of continuous lipid bilayers that self-assemble by spiral wrapping, resulting in relatively rigid cylindrical structures with the drug.

Among the remaining clinical trials retrieved, two are undergoing phase III: a cholesterol-rich, protein-free nanoemulsion of paclitaxel, that resemble low-density lipoproteins and can be IV administered for the treatment of atherosclerosis, and lipid NP with *patisiran* (ALN-TTR02), a siRNA to treat hereditary transthyretin (TTR) induced amyloidosis, made with an optimized ionizable cationic lipid, DLin-MC3-DMA (Kulkarni et al., 2018).

Short interfering RNAs (siRNAs) are 19-23 base pairs double stranded RNAs that are part of the family of small non-coding regulatory RNAs (sncRNA). In Fire et al. (1998) described the gene silencing regulation mechanism of siRNA in *Caenorhabditis elegans*, unveiling what later would become a major change in human therapy approaches. This suppression mechanism, named RNA interference (RNAi), is a normal mechanism of gene expression control involving regulation of mRNA translation and degradation via the binding of short strands of homologous RNA generated by the Dicer enzyme (Reynolds et al., 2004).

Therefore, siRNA presents as an appealing therapeutic tool to suppress gene expression, that can be used to silence aberrant endogenous genes (as in cancer diseases) or to knockdown genes that are essential to the proliferation of infectious organisms (Whitehead et al., 2009). However, in order to become a successful tool for human therapy, siRNA might be administered as an exogenous RNA product, thus representing a drug delivery challenge (McManus and Sharp, 2002). Despite the use of cationic lipids is the natural approach to encapsulate negatively charged biomolecules like nucleic acids, lipids with a *permanent* positive charge tend to form complexes with nucleic acid polymers with limited or no *in vivo* utility due to their size (*ca.* 1 μm of diameter), instability, positive surface charge, and toxic side effects (Cullis and Hope, 2017). Ionizable cationic lipids, on the other hand, allow achieving high loading efficiencies for RNA/DNA molecules in small (<100 nm) vesicular systems, with low surface charge (almost neutral) and less toxicity issues compared with cationic NPs, as discussed in the previous section.

In general, these lipids present an amine group with a pKa value less than 7, a characteristic that allows them to be positively charged at low pH values, thus achieving efficient encapsulation of negatively charged polymers at acidic pH, but also to exhibit a relatively neutral surface at physiological pH values (Kulkarni et al., 2018). As mentioned earlier, a recent research breakthrough on lipid NPs encapsulating siRNA was the 21 base pairs siRNA drug *patisiran* to treat TTR amyloidosis, a multisystemic disease causes by misfolded TTR, that affects nerves, heart, and the gastrointestinal tract (Adams et al., 2017). *Patisiran* lipid NPs reduced amyloidogenic protein expression of the mutated TTR: previous phase II results (NCT01617967) showed an 80% decrease on TTR levels in serum. The efficacy results of patisiran constitute a milestone in the field and led to the approval of the first targeted RNA-lipid NP- based therapy in August Pastor et al. (2018).

Another kind of RNA therapy is not directed to *silence* a given gene but rather to *express* its product, a therapeutic protein. Dimitriadis was the first to achieve expression of rabbit globin on mouse spleen lymphocytes through the delivery of rabbit reticulocyte 9S mRNA through liposomes (Dimitriadis, 1978). This RNA therapy involves much larger mRNA molecules than siRNA (1–15 kb, 300-5000 kDA vs. 14 kDa). Therapeutic approaches may include immunotherapy through mRNA of antibodies, protein expression to supply the product of a defective or missing gene, and *cellular reprogramming* through growth or transcription factors that modulate cellular metabolism. These kinds of treatment can be considered improvements over direct protein administration which face numerous problems like enzyme degradation or misfolding (Kowalski et al., 2019).

The phase I clinical trial of the biological product named mRNA-2416, which encodes for OX40L, the ligand of the T cells co-stimulator tumor necrosis factor receptor superfamily member 4 (TNFRSF4; OX40). According to the NCI drug dictionary, expressed on the cancer cells membrane, OX40L binds to its receptor on T cells to activate a signaling pathway that leads to an increased cytokine production, thus inducing proliferation of lymphocytes and subsequent death of surrounding cancer cells. As in the siRNA systems, this mRNA is formulated in an ionizable lipid-based NP for intratumoural injection to patients with relapsed/refractory solid tumor malignancies or lymphoma.

Another clinical trial (NCT03739931) on lipid nanoparticles for mRNA delivery to solid tumor malignancies or lymphoma is currently recruiting patients. In this case, NPs are used for the intratumoral administration of mRNA-2752, that encodes OX40L, IL-23 and IL-36G. The co-administration of interleukins is thought to potentiate the anticancer effect by activating an inflammatory response at the tumor site (Bauer et al., 2019).

In June 2020, started two phase I clinical trials of ARCT-810, a mRNA therapy for Ornithine Transcarbamylase (OTC) deficiency, a genetic disorder produced by a mutation on the X chromosome. OTC enzyme is involved in the nitrogen metabolism, and the lack of this protein results in high blood ammonia levels that might lead to seizure and coma state in untreated patients. The current treatment is a low protein diet and ammonia scavenging medication, with future insights on liver transplantation (Peng et al., 2020). ARCT-810 is formulated in a novel pH-responsive lipid delivery system named LUNAR®, that aims at superseding the lack of native OTC providing a complete copy of its mRNA, thus restoring enzyme levels to establish a regular urea cycle. Preclinical data on a murine model showed full expression of the protein (Perez-Garcia et al., 2019).

In view of the current situation regarding the SARS-CoV-2 pandemic scenario, a recent clinical study respecting a mRNA-based vaccine has been initiated. The vaccine consists of a lipid nanoparticle encapsulating mRNA-1273 which encodes for the full length prefusion stabilized spike protein of the virus (SARS-CoV-2 spike glycoprotein). After intramuscular administration, mRNA-1273 translates in the myocytes’ cytoplasm. Spike protein is released from the cell and captured by macrophages, dendritic cells, and other immune cells initiating the immune response (Wang et al., 2020).

Leaving aside the CAMB and Oxiconazole formulations, the described nanovehicles share several common characteristics. They are non-viral delivery systems derived from classic liposomes, where the introduction of (permanent or transient) positively charged lipids that have strong electrostatic associations with RNA polymers provides larger payloads of this biomolecules (Cullis and Hope, 2017). Furthermore, most of them are intended for parenteral administration, with the liver as target organ. The fact that siRNA therapy is confined to the liver is a consequence of the tendency of these smaller and more homogeneous analogs of lipoplexes to accumulate in the liver (Wittrup and Lieberman, 2015; Kulkarni et al., 2018).

## Current Challenges and Limitations of Solid Lipid Nanoparticles

The information presented in the previous section reveals that, aside from cosmetic/dermatological applications (Müller et al., 2014), there are currently no *classical* SLN/NLCs in clinical evaluation stages, so their early entry into the market would not be expected. Liposomes that have entered the market in recent years are the result of more than 50 years of research. Similarly, we could think that there are still years to come before SLN/NLC enter the pharmaceutical market. A proof that they are still in their initial stages of research can be seen in the publications reviewed here, a large percentage of which are technological (and not disease) focused research, and usually lacking of a rationale cost/benefit analysis, a characteristic of the initial stages of any research.

A great number of SLN/NLC reports are based on experimental drugs with no approved therapeutic indications, like curcumin, rhein or quercetin, while many others encapsulate pharmaceutical ingredients for which formulations with good therapeutic performance are currently available with low associated costs, such as famotidine, carvedilol, metformin, ibuprofen, dexamethasone, aliconazole, and many others. But it is not all bad news. In the case of RNA or DNA therapies, the use of nanovehicles results essential, since parenterally administered “naked” nucleic acids fail to reach therapeutic levels in target cells. They are rapidly degraded in biological fluids (and excreted by the kidneys) and, if they get to the target tissue, cannot penetrate into the cells (Cullis and Hope, 2017). Therefore, whatever the associated cost, it is compensated by the possibility of having this type of therapies in the market.

**FIGURE 6:**
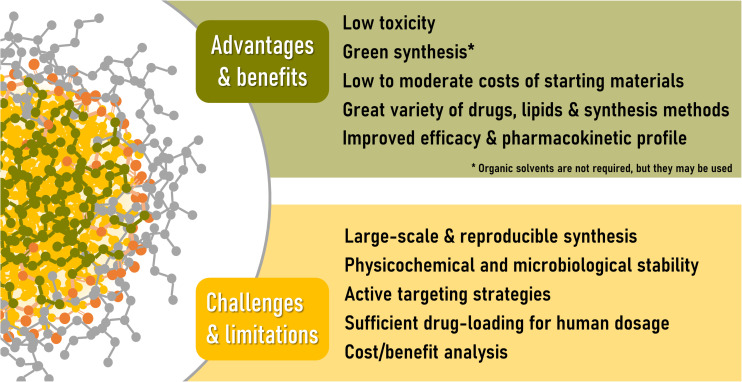
Advantages, current challenges and limitations of working with solid lipid nanoparticles for drug delivery applications.

Taken altogether, current results on pharmacological application of SLN/NLC show good perspectives. They have proven to be safe and versatile drug delivery systems capable of improving the efficacy and pharmacokinetic profile of the encapsulated drugs and, as discussed in the previous sections, many are the therapeutic fields that can be benefited from the use of these nanocarriers. Large-scale manufacturing processes, sterilization, tailoring strategies and stability issues are some of the challenges that need to be overcome before lipid nanoparticles may become commercially available products, with approved therapeutic indications (Figure 6).

## Conclusion and Final Remarks

In the last decade, SLNs and nanostructured lipid carriers have attracted much attention as potential drug delivery (nano)systems. Their major advantage is possibly the use of biocompatible, environmental-friendly constituents and preparation methods. Based on their size and biodegradable nature, most of the nanocarriers in this category fall within the low risk class (class I) from the nanotoxicological classification system suggested by Keck and Müller. It should be noted, though, that careful clinical and environmental safety assessment should be performed before advancing these systems to massive production and commercialization. Development of standardized procedures to assess potential risks of exposure to nanomaterials are urgently needed, as well as the correspondent regulatory framework.

As for other nanosized drug delivery systems, cancer therapy is the most frequent area of research where SLNs are applied, which may reflect both the vast levels of funding in the area but also the suitability of nanocarriers for the delivery of antineoplastic agents, mainly due to the passive and active targeting posed by cancerous cells and tissues. Nevertheless, there are many therapeutic fields that can benefit from the application of lipid NPs, as discussed for the case of antibiotics and CNS drugs.

Given that the oral administration route is the most convenient and accepted one for conventional medications, the fact that nanocarriers (including SLNs) administered through the oral route are not absorbed extensively, it may seem a daunting scenario for the advancement of this technology. However, this may not be true for the particular case of lipid NPs, since they have demonstrated their ability to increase the BA of drugs administered orally, a critical point when it comes to (the increasingly numerous) drugs with very low solubility in water.

Unfortunately, more time and financial resources are needed for SLN/NLC to prove its therapeutic value in real scenarios. For now, the scarcity of SLNs that have reached clinical trials indicates that at least some years will go by before these technologies land to the pharmaceutical market.

## Author Contributions

MR conceived the structure of the manuscript and the search criteria and completed, revised, and approved the manuscript. SM performed the bibliographic search and generated the database. SM and GM analyzed the database and wrote parts of the manuscript. All authors contributed to the article and approved the submitted version.

## Conflict of Interest

The authors declare that the research was conducted in the absence of any commercial or financial relationships that could be construed as a potential conflict of interest.

## Footnotes

1 www.cancer.gov
